# Frailty: new horizons in older patients needing surgery

**DOI:** 10.1308/rcsann.2026.0068

**Published:** 2026-07-01

**Authors:** I Wilkinson

**Affiliations:** Surrey and Sussex Healthcare NHS Trust, UK

We are in the middle of a period of considerable demographic and social change, one that is going to require a reformatting of the training and delivery of healthcare worldwide. Surgical techniques are becoming ever more refined, and more minimally invasive and robotic procedures are being undertaken. We have a greater digitalisation of our systems and artificial intelligence (AI) will revolutionise several aspects of healthcare. On the other hand, at the same time, our population is growing, living longer and ageing. The proportion of people aged over 85 years in the UK is set to double in the next 20 years.^1^ With that comes a significant increase in the conditions that affect older people, and a concomitant change in patients receiving elective and non-elective care from surgical teams.

## Age vs frailty

As we age, our organs’ ability to function maximally reduces year on year (e.g. renal perfusion declines by about 1% per year from age 40 and VO_2_ max about the same but from around age 25).^2,3^ These physiological changes are a result of the ageing process. We all age at different rates, however, and so a more detailed exploration of the effects of ageing (as opposed to chronological age) in an individual is needed. This is essentially the study of frailty.

The term ‘frailty’ has been used in the English language since the 14^th^ century but only in the past 25 years has clear, focused attention been paid to the causes, physiology and impact of frailty. In 2001, Fried and her colleagues identified a phenotypic method for the identification of frailty, with three or more items showing the presence of frailty and the negative outcomes associated with this state (Table 1).^4^ Of these, gait speed is especially helpful and easy to measure, with a gait speed of <1 m/s having poorer healthcare outcomes.

**Table 1:** Fried frailty phenotype^4^

Item	Type of measure	Criteria
Weight loss (unintentional)	Self-reported	>4.5 kg in past year
Exhaustion (self-reported)	Self-reported – ‘trouble getting going’	Poor endurance and energy
Weakness (grip strength)	Dynamometer	In lowest 20% for sex and body mass index
Slowness (walking speed)	Timed 5 m walk	In slowest 20% for sex and height
Physical activity (reduced)	Self-reported – calories expended	In lowest 20% of kcal expended per week
*Significance*: • 1–2 criteria = intermediate frailty (sometimes called pre-frail); increased risk of becoming frail over next few years • ≥3 criteria = frailty; increased risk of falls, worsening mobility or difficulty with activities of daily living, hospitalisation and death		

When viewed through this lens, frailty stems from the development of sarcopenia – the progressive loss of muscle mass and strength (particularly fast-twitch [type 2] muscle fibres). These types of muscle are especially key for common activities such as our mobility, coughing and ability to stand up. Various mechanisms then lead to a negative energy balance, increased muscle loss and a low level of chronic inflammation (so called inflamm-ageing).

**Figure 1:**
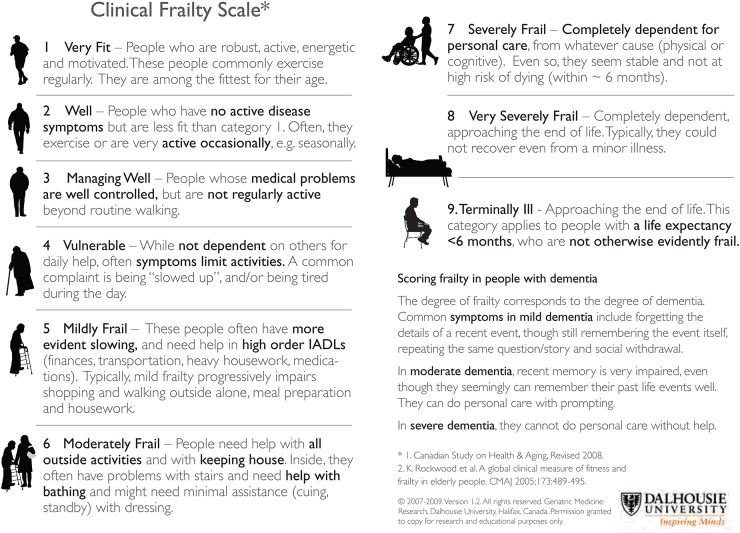
Clinical frailty scale^6^

In 2007 Rockwood and Mitnitski focused on frailty being an accumulation of deficits leading to an increased vulnerability to stresses placed on the patient’s physiological system (a loss of ability to maintain homeostasis with a stressor).^5^ This led to the production of two important tools that are widely used today: the clinical frailty scale (CFS), which is probably the best known (Figure 1),^6^ and the electronic frailty index (eFI), which was updated in 2025 as the eFI2, developed to be integrated into UK primary care records.^7^ In essence, the tool comprises a list of conditions that are more common with age and confer a negative outcome. The patient is scored against this weighted list. The cut-off score is <0.16 for mild frailty, <0.24 for moderate frailty and ≥0.24 for severe frailty (i.e. approximately a quarter of all conditions on the list).

Importantly, age is not part of the definition of frailty when using the deficit accumulation or phenotypic methods of identification. Frailty is consequently not the same as chronological age and some individuals live long without becoming frail while others develop significant frailty at a younger age. When an individual is frail, any given illness will affect them more severely as their usable reserve in physiological function is lower. As we age, frailty becomes much more common and this explains data, including SNAP3 (the third Sprint National Anaesthesia Project),^8^ that highlight that even mildly frail patients face a much higher risk of adverse events. Nevertheless, despite clear guidance from the Centre for Perioperative Care and the British Geriatrics Society as well as a developing evidence base, a considerable implementation gap and variation in practice remain (as shown by Kler *et al* in this issue of the *Annals*).^9^

## Frailty identification

A patient only has a diagnosis when it is named by a clinician; until then, they have a collection of signs and symptoms. Proactive and systematic frailty identification is therefore key, and the CFS and eFI make this easier. The CFS can be administered by anyone and there is an excellent CFS app, with a clear set of questions, which takes around 30 seconds to complete, giving an accurate repeatable assessment. The tool is intended to establish the baseline health state (i.e. how the patient was before they became ill).

The eFI is integrated into many general practice systems and may be increasingly included in referrals to services. In addition, the National Institute for Health and Care Excellence advocates the use of the FRAIL acronym to structure frailty assessment and care (Table 2).^10^

**Table 2:** The FRAIL acronym advocated by the National Institute for Health and Care Excellence^9^

Step	Example
**F**ind	Systematically identify individuals who may be living with frailty (e.g. clinical frailty scale or electronic frailty index).^6,7^
**R**ecognise	Acknowledge frailty as a distinct long-term health condition rather than an inevitable part of ageing. Recognition includes identifying ‘frailty syndromes' such as falls, sudden immobility or new-onset confusion (delirium).
**A**ssess	Conduct a comprehensive geriatric assessment.
**I**ntervene	Implement targeted clinical and supportive actions.
**L**ong-term	Transition to sustained, long-term support and follow-up. View frailty as a chronic condition with a personalised care and support plan that is shared across primary, secondary and social care teams

For surgical practice, frail patients will present both in the elective and non-elective settings. Electively, the CFS or eFI will identify patients and needs integration into processes and systems for all patients aged over 65 years to be assessed. In the future, AI-based systems may identify sarcopenia from routine computed tomography, giving an opportunity for a much earlier intervention and the potential to stave off frailty. In the non-elective setting, patients will often present with a frailty-defining illness (such as a fall with a fracture, or delirium following an infection or a procedure).

## Frailty management

The ongoing management of a frail patient is via a comprehensive geriatric assessment with surgical teams being a key element of this. In medicine, much management is based on population-level data (e.g. treatment of hypertension reduces the risk of heart disease) but as patients become frailer, a personalised approach is needed. Asking what matters most to that patient is a good place to start.

Comprehensive geriatric assessment is a process of patient assessment through several domains that include functional, physical, psychological, environmental and social aspects. A problem list is then created, and a multiprofessional team develops a personalised management plan for and with the patient. It is a process, not a one-off intervention, and there is a good evidence base for this in surgical practice. More specifically, a frail patient’s management is management of both the underlying sarcopenia (via protein intake and exercise), their other associated conditions and treatments, and the functional and social impact of all of it together.

Orthogeriatrics is the most embedded of these integrated models, leading to around a halving of 30-day mortality following neck of femur fractures from the creation of the National Hip Fracture Database in 2007 until now.^11^ Similar evidence and some datasets are present for geriatric integration into other surgical teams (the National Emergency Laparotomy Audit, for example) but uptake is, as yet, less widespread.

## The future of frailty care

Despite the evidence, there have not been commensurate increases in the numbers of trainees in geriatric medicine for all surgical and oncological teams to have the same model of service. This also comes at a time when medical specialists are seeing more geriatric medicine integration and involvement in the newer services of same-day emergency care, urgent care response teams and community-led multidisciplinary teams. Indeed, the drive to a greater neighbourhood model of care in the government’s 10 Year Health Plan for England^12^ is likely to take geriatrician time out of acute hospitals into the neighbourhood teams, seeing patients closer to their homes.

As highlighted by the Chief Medical Officer for England, Professor Sir Chris Whitty, all members of our workforce therefore need to develop the generalist skills to manage frail older patients.^13^ This is now rapidly becoming ‘business as usual’ for our healthcare, with specialised geriatric medicine services being there for the most complex cases. Patient and operative choice has never been more important.

## Decision making with frail patients: what matters most?

Owing to the rising numbers of older patients living with frailty, there has never been a better time to focus on the patient and operative choices. Making the correct decisions with patients in the preoperative period will substantially reduce the risks in the perioperative and postoperative settings. Asking what matters most to them is very helpful in this regard. Not all patients will want or need surgery to achieve their priorities and wishes. Being clear about these wishes is a key element in the initiation of an anticipatory or advance care plan.

Obviously, the first choice is about the means and method of investigation, and once a diagnosis is made, there are potential differences in management options. Thinking about the impact of a patient’s frailty during these stages is common among individual clinicians but this is not always systematic. There may be scope to optimise patients medically prior to surgery, although time windows might be small and outcomes may be better with a delay to surgery for this to happen (with appropriate patient consent). This is being explored in several ‘surgical pause’ trials. Some procedures (e.g. cardiac surgery and arthroplasty) have also been shown to improve overall frailty by enabling patients to exercise more, and reducing long-term deconditioning together with its associated functional decline and dependency.

## Prehabilitation and deconditioning

If a healthy patient is in bed for a week, they can lose up to 7% of their muscle mass; this is likely to be much more in frail patients.^14^ This often has a considerable effect on their ability to recover and return home to their usual residence. We need to develop systems that stop this from happening. Prehabilitation is now well accepted as a standard of care to improve outcomes and there are a number of digital solutions to assist with this.

Nursing and medical care following admissions needs to aid us here too. Catheters are often not needed as much as we use them. Management strategies that enable more rapid weight bearing and less mobility restriction (e.g. hindfoot nails in some ankle fractures or the recent trials looking at less immobilisation following cervical spine fractures) can help – albeit with careful patient selection. Improved identification of delirium (using the 4AT test), reduced delirium precipitation (e.g. through regional anaesthesia) and better management of early delirium with less reliance of unproven pharmacological restraint help maintain mobility, and reduce the length of delirious episodes.

## Conclusions

Surgeons will be investigating, managing and operating on increasing numbers of older patients. Some of these will also be living with high levels of frailty. The systematic identification of these patients and taking the time to explore what matters most to them will allow a degree of pragmatism around their management. The involvement of geriatricians can be beneficial but the geriatric medicine workforce is currently being stretched in many directions and so generalist skills in the management of frailty will be needed by all.
